# Clinical and genetic characteristics of Chinese Duchenne/Becker muscular dystrophy patients with small mutations

**DOI:** 10.3389/fnins.2022.992546

**Published:** 2022-11-07

**Authors:** Siyi Gan, Shulei Liu, Haiyan Yang, Liwen Wu

**Affiliations:** Neurology Department, Hunan Children’s Hospital, The School of Pediatrics, Hengyang Medical School, University of South China, Changsha, China

**Keywords:** Duchenne muscular dystrophy, Becker muscular dystrophy, small mutations, clinical characteristics, genetic characteristics

## Abstract

**Background:**

Duchenne muscular dystrophy (DMD) and Becker muscular dystrophy (BMD) are amongst the inherited neuromuscular diseases with the highest incidence. Small mutations are less common and therefore have been poorly studied in China.

**Materials and methods:**

The clinical data of 150 patients diagnosed with DMD/BMD by genetic analysis in Hunan Children’s Hospital from 2009 to 2021 were analyzed. The patients were followed up for an average of 3.42 years and their clinical characteristics were collected. Loss of ambulation (LOA) was used to evaluate the severity of disease progression. The correlation among clinical features, different variants, and glucocorticoid (GC) treatment was analyzed by Cox regression analysis.

**Results:**

150 different variants were detected in this study, including 21 (14%) novel mutations, 88 (58.7%) non-sense mutations, 33 (22.0%) frameshift mutations, 22 (14.7%) splicing mutations, and 7 (4.7%) missense mutations. Single-exon skipping and single- or double-exon (double/single-exon) skipping strategies covered more than 90% of patients with small mutations. A case with frameshift mutation combined with Klinefelter’s syndrome (47, XXY) and another one with missense mutation combined with epilepsy was found in our study. *De novo* mutations accounted for 30.0% of all patients. The mean onset age was 4.19 ± 1.63 years old, and the mean diagnosed age was 5.60 ± 3.13 years old. The mean age of LOA was 10.4 years old (40 cases). 60.7% of them received GC treatment at 7.0 ± 2.7 years old. The main causes of complaints were muscle weakness, high creatine kinase (CK), motor retardation, and family history. The risk factors of LOA were positive family history (HR 5.52, CI 1.26–24.18), short GC treatment duration (HR 0.54, CI 0.36–0.82) and frameshift mutation (HR 14.58, CI 1.74–121.76). DMD patients who treated with GC after 7 years old had a higher risk of earlier LOA compared to those receiving treatment before the age of 7 (HR 0.083, CI 0.009–0.804). Moreover, an earlier onset age, a higher CK value, and a larger LOA population were found in the DMD patients compared to the BMD ones. Finally, the locations of the most frequent mutation were in exon 70 and exon 22.

**Conclusion:**

In conclusion, 150 small mutations were identified in this study, and 21 of them were discovered for the first time. We found the hotspots of small mutations were in exon 70 and exon 20. Also, the analysis showed that positive family history, frameshift mutation, short duration of GC treatment, and delayed GC treatment resulted in earlier LOA for the DMD patients. Taken together, our findings complement the mutation spectrum of DMD/BMD, benefit us understanding to the DMD disease, and lay foundations for the clinical trials.

## Introduction

Duchenne muscular dystrophy (DMD) and Becker muscular dystrophy (BMD) are neuromuscular recessive disorders due to the X-linked mutation in the dystrophin gene (Tuffery-Giraud et al., 2009). DMD is considered to be the most destructive category due to the complete loss of the dystrophin protein. Progression of the disease eventually leads to loss of ambulation (LOA) around age 13. BMD with a later onset and a slower progression than DMD (Duan et al., 2021). In general, the average age of onset of BMD is 12 years, and overall life expectancy is longer (Bello et al., 2016). Recent studies have greatly improved our understanding of pathogenesis. Multidisciplinary care guidelines for DMD have been established, covering all aspects of access to genetic diagnosis and management of the disease (Duan et al., 2021).

Approximately 60–70% of mutations in patients with DMD are deletions and have therefore become a popular topic of research (Dzierlega and Yokota, 2020). While small mutations, including non-sense mutations, missense mutations, frameshift mutations, and splicing mutations, accounted for 20% of the genetic data, remaining largely unknown. Severe DMD is caused by non-sense or frameshift mutations in the DMD gene, whereas its milder form of BMD is caused by in-frame deletions/duplications or missense mutations (Juan-Mateu et al., 2013; Okubo et al., 2020). There are also some patients with non-sense mutations who exhibit the BMD phenotype, mainly due to skipping exons containing non-sense mutations, resulting in in-frame deletion (Okubo et al., 2020). Some scholars have proposed that the location of non-sense mutations can predict the severity of phenotypes (Torella et al., 2020). Therefore, the molecular and clinical study of small mutations remains a complex problem.

Previous studies have analyzed the genetic characteristics of small mutations and identified the DMD/BMD phenotypes, but relatively few have evaluated the response of those patients to glucocorticoid (GC) treatment. Although many patients with small DMD mutations are distributed in various regions of China, they remain understudied. In this study, 150 patients with all minor mutations were enrolled to further analyze the correlation between clinical and genetic characteristics and to comprehensively investigate the effectiveness of GC treatment.

## Materials and methods

### Patients

Duchenne muscular dystrophy/Becker muscular dystrophy was clinically diagnosed based on clinical symptoms, family history, electromyography (EMG) data, and creatine kinase (CK) levels. In all probands, the diagnosis was confirmed by identification of family members with severe muscular atrophy and loss of ambulation before age 20. And CK levels above 400 U/L was defined as high CK. All patients with DMD small mutations were further confirmed by genetic testing, excepting those without large fragment deletions and genetic testing data. All participants provided informed consent for molecular analysis and participated in the study, which was approved by the hospital’s ethics committee.

A team of neuromuscular disease specialists was formed based on the age of symptom onset, disease progression, family history, and the age of LOA, and all patients were classified into four categories: Duchenne muscular dystrophy (DMD, LOA <13 years old); Becker muscular dystrophy (BMD, LOA ≥16); Intermediate muscular dystrophy (IMD, 13 years ≤ IMD < 16 years) and undetermined. Patients with motor function severity between DMD and BMD were classified as IMD. (Juan-Mateu et al., 2015; Andrews et al., 2018). The rest of the patients beginning to experience muscle weakness before age five was defined as having DMD, and with basically normal motor function or very mild muscle weakness after age five were defined as having BMD (Wang et al., 2019). We were unable to assign asymptomatic and young patients to phenotypic categories.

The study included five IMD patients, four of whom lost ambulation between 13 and 14 years old, and the other one patient is now 16 years old. Although the onset age is early, he had difficulty in going up and down stairs, squatting and standing up, and did not lose ambulation. Therefore, he was identified as an IMD patient.

### Genetic analysis

Blood samples with negative multiplex ligation-dependent probe amplification (MLPA) results were then checked for small-scale mutations by next-generation sequencing (NGS) using an Illumina HiSeq 2000 platform (Illumina, San Diego, CA, United States) or Sanger sequencing as previously described, which was used to detect whether the probands’ mothers carried the DMD mutation.

### Statistical analysis

IBM SPSS Statistics software, version 24.0, was used for statistical analysis of the clinical data. All counting data are presented as the number of cases or percentages. The measurement data with a normal distribution are presented as the mean ± standard deviation, and the measurement data with a non-normal distribution are presented as the median. Chi-square test was used for enumeration data. Cox proportional hazards regression was used to estimate the effect of individual variables on age at LOA. Statistical significance was set at *p* < 0.05.

## Results

### Clinical features

#### General condition

The entire cohort of 150 patients was enrolled in our study with a mean follow-up of 3.42 years and an average age of 8.9 years old. All patients came from central and southern China, and there were no significant differences in ethnic or geographic distribution. The average age of onset of symptoms was 4.19 years old. The main reasons for referral were muscle weakness found by family members, including difficulty in climbing stairs, squatting, standing up from squatting, slow running or heel failure to land after squatting (40.0%, *n* = 60). Additional clinical symptoms include high CK values, motor retardation, and family history. CK levels increased, ranging from 480.7 upto 43,716.0 IU/L, with an average of 13,108.5 IU/L (Table 1).

**TABLE 1:** Clinical characteristics of Duchenne muscular dystrophy/Becker muscular dystrophy (DMD/BMD) with small mutation.

Characteristics	All patients	DMD	BMD	*P*-value
Age (years)	8.9 ± 3.7	9.3 ± 3.3	9.3 ± 2.6	−
Age of symptom onset (years)	4.19 ± 1.63	3.7 ± 1.3	6.6 ± 1.0	<0.0001
Age of diagnosis (years)	5.60 ± 3.13	5.6 ± 3.0	7.1 ± 2.6	0.019
Age of LOA (years/%)	10.4 ± 2.1	35 (87.5%)	1 (2.5%)	0.008
GC treatment patients (%)	89 (59.3%)	72 (80.8%)	9 (10.1%)	0.064
GC treatment initiation (years)	7.0 ± 2.7	7.0 ± 2.6	7.0 ± 2.4	0.962
Serum CK level	13,108.5 ± 8,690.2	13,465.7 ± 9,424.0	8,510.0 ± 3,762.6	0.015
New mutations (%)	45 (30.0%)	29 (64.4%)	8 (17.7%)	−

DMD, Duchenne muscular dystrophy; BMD, Becker muscular dystrophy; LOA, loss of ambulation; GC, glucocorticoid; CK, creatine kinase.

The average age of genetic diagnosis was 5.60 ± 3.13 years old, and 48.7% of them were diagnosed before 5 years old. Patients with non-sense mutations were diagnosed at elder age (5.90 years old), and their loss of ambulation delayed (10.8 years old) than those with other small mutations. 16.6% of them (25) had a positive family history of the disease. During the period of this study, 40 patients experienced LOA, and the mean age was 10.4 ± 2.1 years old (5.5–17). There were no deaths during follow-up. Clinical characteristics showed that, compared with BMD patients, the symptoms onset and diagnosis of DMD patients were at an earlier age, and they often had higher LOA ratios and higher CK levels (*p* < 0.05). The results are shown in Table 1.

#### Glucocorticoid treatment

In the group of this study, 89 boys (59.3%) received GC treatment, and 61 boys with an average age of 7.79 ± 4.36 years old had never received GC treatment. The mean age receiving GC treatment for the first time was at 7.0 ± 2.7 years old (1.2–13.7 years). The average prescribed dose was 0.57 ± 0.19 mg/kg/d prednisolone or 0.62 ± 0.21 mg/kg/d deflazacort. 79 of 90 patients treated with GC were followed up for over 1 year, and the maximum and mean duration of GC treatment was respectively 11.5 years and 2.75 years. The mean age of LOA was at 10.9 years old (*n* = 91) for those receiving GC treatment, and at 9.8 years old for non-users (*n* = 59). The proportion of GC utilization non-sense mutations, frameshift mutations, splicing mutations, and missense mutations were 67.0, 48.5, 100, and 28.6%, respectively. The main side effects of GC treatment are cushingoid features, abdominal obesity, delayed growth, cataracts, and elevated blood pressure.

### Molecular characteristics

**FIGURE 1:**
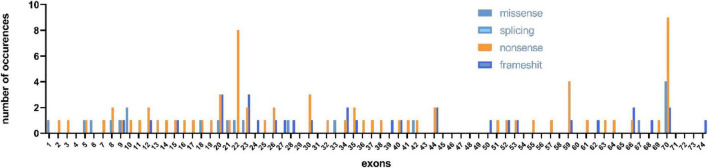
Distribution of exons of small mutations in different mutation types.

**FIGURE 2:**
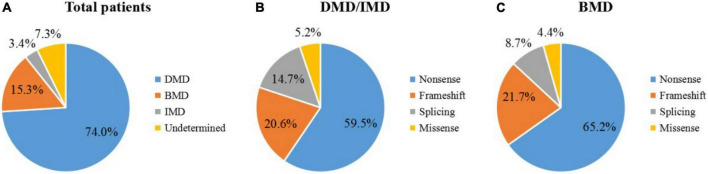
Distribution of small mutations. The ratios of mutation types in all patients **(A)**, DMD/IMD **(B)**, and BMD **(C)**.

In our study, 150 male patients were identified by DMD gene sequencing (111 DMD, 5 IMD, and 23 BMD), and the other 11 patients could not be categorized precisely because of their young age. Among the mutation genotypes, non-sense mutations, frameshift mutations, splicing mutations and missense mutations accounted for 58.7 (88/150), 22.0 (33/150), 14.7 (22/150), and 4.7% (7/150), respectively. Non-sense mutations were the most common type of mutation (60.3%, 53/88), and the most common substitution was C > T. Among the frameshift mutation, there were 19 small deletions (57.5%), and 14 small insertions (42.4%). No other exon hot spots of mutation were identified except the exon 70 and 22 (Figure 1). The frequency of mutation types varies by clinical phenotype (Figure 2). There was no obvious same distribution pattern among different types of small mutations. Of the 150 mutations, 21 novel mutations in 21 unrelated families were first reported in our study (Table 2), and these novel mutations were found in 30.0% (45/150) of patients.

**FIGURE 3:**
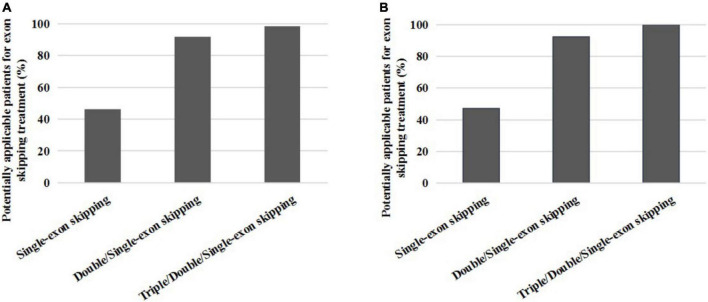
The proportion distribution of potential small mutations single and multiple exons skipping treatments in all patients **(A)** and DMD/IMD **(B)**.

**TABLE 2:** The 21 novel mutations diagnosed by next-generation sequencing (NGS) sequencing technology.

Position	Type of mutation	Nucleotide change	Mutation at protein level	Pathogenesis	Carrier status of mother
Exon 18	Frameshift	c.2292 + 1G > A	**/**	LP	YES
Exon 20	Frameshift	c.2591delC	p.T864Kfs 7	P	/
Exon 59	Nonsense	c.8785delA	p.I2929	P	YES
Exon 59	Frameshift	c.8776_8788del13	p.Q2926Mfs 26	LP	YES
Exon 50	Frameshift	c.7265delC	p.A2422Efs 5	P	YES
Exon 70	Nonsense	c.10084C > T	p.Arg3362Ter	P	YES
Exon 57	Nonsense	c.8114T > A	p.L2705X	VUS	YES
Exon 68	Frameshift	c.9877delG	p.Val293Cysfs 37	P	YES
Exon 63	Nonsense	c.9258delC	p.Met3088	P	YES
Exon 35	Nonsense	c.4984C > T	p.Arg1662Ter	LP	YES
Exon 44	Frameshift	c.5622_5623insTA	p.Glu1874Glu fs X2	VUS	YES
Exon 30	Frameshift	c.4186delT	p.Tyr139611efsTer22	LP	YES
Exon 7	Nonsense	c.583A	p.R195X	P	YES
Exon 53	Frameshift	c.7755del G	p.Trp2585Cysfs 12	P	YES
Exon 67	Splicing	c.9807 + 3A > C	**/**	VUS	YES
Exon 39	Frameshift	C.5569_5570delAA	**/**	P	YES
Intron 45	Splicing	c.6614 + 1G > C	**/**	P	NO
Exon 35	Frameshift	c.4909_4910insGG	p.A1637Gfs 3	P	**/**
Exon 9	Frameshift	c.905_908dup	p.Gln303 HisfsTer10	P	NO
Intron 66	Splicing	c.9649 + 3A > C	/	VUS	NO
Exon 23	Missense	c.2996T > C	p.L999P	VUS	YES

We found that single-exon skipping, single or double-exon (double/single-exon) skipping, and single, double, or triple-exon (triple/double/single-exon) skipping covered 46.3, 91.9, and 98.4% of patients with small mutations, respectively. After removing BMD and unclassified patients, we found that single exon, double/single exon, and triple/double/single exon skipping covered 47.2, 92.5, and 100% of patients with DMD small mutations, respectively (Table 3).

**TABLE 3:** Multivariate Cox regression analysis of survival time in children with Duchenne muscular dystrophy (DMD) treated with glucocorticoid (GC).

Cox regression factor	HR	*P*-value	95% CI
Symptom onset- diagnosis delay	0.98	0.934	0.64–1.49
Diagnosis-GC treatment delay	1.20	0.333	0.82–1.77
GC type	0.58	0.627	0.70–4.98
GC dose	0.14	0.180	0.008–2.48
Positive family history	5.52	0.023	1.26–24.18
Time of GC treatment initiation	0.08	0.032	0.009–0.80
GC treatment duration	0.54	0.004	0.36–0.82
Non-sense mutation	1.79	0.264	0.64–5.00
Frameshift mutation	14.58	0.013	1.748–121.76

CI, confidence interval; HR, hazard ratio; GC, glucocorticoid.

One patient with a frameshift insertion mutation (exon 35: c.4909_4910insGG; chrX-32383252; p.A1637Gfs*3) is now 6 years old and has only slightly difficulty in walking up and down stairs, and significantly increased CK (12,013 U/L). This mutation (C.4909_4910INSGG) has not been reported in the HGMDpro database. According to the American College of Medical Genetics and Genomics (ACMG) guidelines, the c.4909_4910INSGG mutation can be rated as a pathogenic mutation. It is noteworthy that we found that the patient’s X chromosome had repeated variation in the next generation sequencing, and clinical feedback showed that the patient’s karyotype was 47, XXY. Thus, he diagnoses as Klinefelter syndrome.

Another patient with a DMD gene missense mutation (exon 23: c.2996T > C; chrX-32486781; p. L999P) combined with epilepsy, showed significantly increased plasma CK levels, an inability to walk up and down stairs, difficulty in standing up in squats, and a positive Gowers’ sign. Electroencephalogram captured the spike wave and spike and slow complex in the Rolandic area. The onset frequency of seizures was once a month, while there were no seizures after 2 years of oxcarbazepine antiepileptic treatment.

### Correlation analysis

To explore the association between loss of ambulation (LOA) and multiple factors, we focused on all patients with DMD and IMD who received glucocorticoid (GC) treatment. Multiple factors include the delay time from symptom onset to diagnosis of the patient, delay time from diagnosis to receive GC treatment, GC type and dose, positive family history, GC therapy initiation time, duration of GC treatment, and small mutation types. Among them, a positive family history is defined as a patient with similar symptoms to their families, such as early muscle atrophy and paralysis around the age of 20. As for GC treatment initiation time, we chose those patients treating GC at average age seven. Cox multivariate analysis suggests that a positive family history, initiation of GC therapy after age 7 years, short duration of GC therapy, and frameshift mutation was main risk factors for LOA (*p* < 0.05). Cox regression parameters for all other covariates did not differ significantly from the reference group (Table 3).

## Discussion

A growing number of studies have revealed large deletions as the major mutation type causing Duchenne, while small mutations have received less attention. We identified small mutations that cause Duchenne/Becker muscular dystrophy using exon-targeted capture second-generation sequencing in a group of Chinese patients. In the past, the analysis of small mutations was mostly based on genetic characteristics, not coupled with clinical characteristics. We analyzed the data of DMD-BMD patients with small mutation who visited the hospital from 2009 to 2021, including the differences in DMD-BMD phenotypes, the risk factors of loss ambulation and the effectiveness of GC treatment, and we paid our attention to DMD gene mutation combined with epilepsy and Klinefelter syndrome. More importantly, we identified 21 novel mutations for the first time, and there are a higher frequency of mutations occurring in exon 70 and 22. The newly discovered mutations in this study provide a theoretical basis for studying the mutation mechanism of the DMD gene and the treatment of DMD/BMD.

In our patients’ general case, the most common reasons for attendance were delayed motor milestones, limb weakness, infection and unintentional elevation of creatine kinase on physical examination, and the age of onset of symptoms, which seemed to be consistent with previous reports (Rao et al., 2014; Liang et al., 2018; Wonkam-Tingang et al., 2020). In addition, some patients might also seek treatment due to short stature, attention-deficit/hyperactivity disorder, developmental delay, and other unnoticed manifestations, which suggest that we should not ignore the possibility of this disease. It has been noted that the reasons for patients receiving treatment include cardiomyopathy, ventilatory dysfunction, scoliosis, and so on. All of these are symptoms of advanced stages of disease (Wang et al., 2011) and are rarely observed in our center. The average age diagnosed as DMD world widely was approximately 4–5 years old (Zhang et al., 2021). The mean age of diagnosis in our study was 5.6 years old, and the average time from symptom onset to diagnosis was 2.91 years, which seems to be delayed than in previous studies reported (Thomas et al., 2022). Global data showed that patients without glucocorticoid treatment lost the ability to ambulate at 10 years old, while those with treatment were at 13 years old (Koeks et al., 2017), and patients in our study were at 10.9 and 9.8 years old, respectively, which was similar to a previous report (Liang et al., 2018). The differences may be explained by the dose of glucocorticoid taken in our cohort and the compliance of the patients taking medication. Of course, studies have shown that patients with or without glucocorticoids will eventually lose ambulation at the age of 13 years old (Sattenapalli et al., 2022).

The efficiency of GC treatments has been confirmed by several studies, which can increase the ambulation and cardiopulmonary function of DMD patients. The age of patients received GC treatments varied from individuals, usually at 4–5 years old (Wang et al., 2019). In our study, 60.7% of the patients received GC treatment, and their mean age was at 7.0 ± 2.7 years old, possibly because their muscle weakness was more pronounced in our cohort at this time. The average dose of prednisone was 0.57 ± 0.19 mg/kg/day, which was lower than the guideline recommendation of 0.75 mg/kg/day. Our study showed that for those treated with GC after 7 years old, they had a higher risk of LOA. The effectiveness of early treatment has also been reported in the literature (Kim et al., 2017). However, our study did not find significant differences in GC type and dose.

Non-sense mutations were the most frequent mutations in patients identified by us (58.7%), which introduced premature termination codons, resulting in truncated form of the protein and further causing severe phenotypes. Of course, an increasing study has reported that a very low rate of BMD cases carried non-sense mutations (Wang et al., 2019). In this study, missense mutations accounted for 4.7% (7/150) of the total small mutations, which were less than in previous studies (Juan-Mateu et al., 2015). Missense mutations have been reported to be associated with increased BMD (Okubo et al., 2020; Tokgün et al., 2022). Exon skipping strategy works well with patients carrying small mutations (Niks and Aartsma-Rus, 2017; Okubo et al., 2020). Half of our patients (approximately 58.6% of all patients) carried non-sense mutations. The application of exon skipping strategy is non-sense mutations, which were also reflected in our patients. Single- and double-exon skipping can theoretically cover 90% of non-sense mutations and small insertions and deletions collectively. Most frameshift mutations in our cohort were found in patients presenting severe DMD or IMD phenotype. Therefore, exon skipping therapy could be used in more severe forms of the disease. Exon 70 and exon 22 mutations were more frequent in our findings. Although most studies have found that small mutations are evenly distributed throughout the DMD gene (Zimowski et al., 2021), hot-spot mutation regions are deserved to be paid more attention, so that more efficient treatment targets can be discovered.

We cannot ignore *de novo* mutations in genetic counseling. In this study, 30.0% (45/150) new mutations were obtained. The mechanism of the new mutations occurring is not fully understood, but germline chimerism could be a possible cause. Therefore, effective and rapid diagnostic methods and systematic pedigree analysis are necessary for genetic counseling for DMD (Kong et al., 2019).

In terms of complications, we found a patient with DMD combined with Klinefelter syndrome, which is a disease caused by a sex chromosome abnormality. It is the most common types of male hypogonadism clinically, and it is one of hypogonadism induced by hypogonadotropism. This type of disease was first reported in 1989 and is very rare (Suthers et al., 1989; Ramesh et al., 1993; Santoro et al., 1998; Xu et al., 2014). Whether a combination of a mutated X chromosome and a normal X chromosome in Klinefelter always leads to BMD needs to be further analyzed, and at the same time, the patient’s phenotype and chromosome source should be considered (Suthers et al., 1989). A patient with frameshift insertion mutation is now 6 years old and has only mild difficulty in walking up and down stairs. Our patient had 2X chromosomes carrying the same mutation in exon 35, so one would expect a DMD phenotype. However, he has a rather mild course and has only limited difficulty walking up and down the stairs at age six. It is possible that the mutation causes a spontaneous skip of exon 35 for some transcripts. As exon 35 is in-frame, this would bypass the mutation and allow production of some dystrophin. Protein analysis would have to be done to confirm this hypothesis. However, similar cases have been described before (Ginjaar et al., 2000). In addition, one of our patients carried a missense mutation (chrX: 32486781, c. 2996T > C, p. L999p) showing epilepsy. Alterations in muscular dystrophy proteins in the brain are involved in an increased risk of epilepsy in Becker and Duchenne muscular dystrophy (BMD and DMD), and no association between mutation sites and prevalence of epilepsy was observed. Changes in muscular dystrophy proteins in the brain are currently linked to an increased risk of epilepsy in DMD, and no associations between mutation sites and epilepsy prevalence have been observed (Pascual-Morena et al., 2022). Missense mutations in this part of the gene are not likely to cause DMD or even BMD. However, it is possible that this mutation disrupts splicing of exon 23 and in that way abolishes dystrophin production. It is also possible that the patient carries a deep intronic mutation that disrupts splicing. Both scenarios can be explored with mRNA analysis.

In summary, small mutant DMD/BMD should be paid more attention to clinical heterogeneity and relatively complex conditions of these patients indicating that individualized treatment strategies are in highly needed in the future.

## Data availability statement

The raw data supporting the conclusions of this article will be made available by the authors, without undue reservation.

## Ethics statement

The studies involving human participants were reviewed and approved by the Ethics Committee of the Hunan Children’s Hospital, Changsha, Hunan. Written informed consent to participate in this study was provided by the participants’ legal guardian/next of kin. Written informed consent was obtained from the individual(s), and minor(s)’ legal guardian/next of kin, for the publication of any potentially identifiable images or data included in this article.

## Author contributions

LW designed and organized the study and revised the manuscript. SG wrote the manuscript. SL and HY collected and analyzed the clinical data and patient. All authors read and approved the final manuscript.

## References

[B1] Andrews J. G. Lamb M. M. Conway K. Street N. Westfield C. Ciafaloni E. (2018). Diagnostic accuracy of phenotype classification in Duchenne and Becker muscular dystrophy using medical record data. *J. Neuromuscul. Dis.* 5 481–495. 10.3233/JND-180306 30320597 PMC6367719

[B2] Bello L. Morgenroth L. P. Gordish-Dressman H. Hoffman E. P. McDonald C. M. Cirak S. (2016). DMD genotypes and loss of ambulation in the CINRG Duchenne natural history study. *Neurology* 87 401–409. 10.1212/WNL.0000000000002891 27343068 PMC4977110

[B3] Duan D. Goemans N. Takeda S. I. Mercuri E. Aartsma-Rus A. (2021). Duchenne muscular dystrophy. *Nat. Rev. Dis. Prim.* 7 1–19. 10.1038/s41572-021-00248-3 33602943 PMC10557455

[B4] Dzierlega K. Yokota T. (2020). Optimization of antisense-mediated exon skipping for Duchenne muscular dystrophy. *Gene Ther.* 27 407–416. 10.1038/s41434-020-0156-6 32483212

[B5] Ginjaar I. B. Kneppers A. L. Anderson L. V. Bremmer-Bout M. van Deutekom J. C. Weegenaar J. (2000). Dystrophin nonsense mutation induces different levels of exon 29 skipping and leads to variable phenotypes within one BMD family. *Eur. J. Hum. Genet.* 8 793–796. 10.1038/sj.ejhg.5200535 11039581

[B6] Juan-Mateu J. Gonzalez-Quereda L. Rodriguez M. J. Baena M. Verdura E. Nascimento A. (2015). DMD mutations in 576 dystrophinopathy families: A step forward in genotype-phenotype correlations. *PLoS One* 10:e0135189. 10.1371/journal.pone.0135189 26284620 PMC4540588

[B7] Juan-Mateu J. González-Quereda L. Rodríguez M. J. Verdura E. Lázaro K. Jou C. (2013). Interplay between DMD point mutations and splicing signals in Dystrophinopathy phenotypes. *PLoS One* 8:e59916. 10.1371/journal.pone.0059916 23536893 PMC3607557

[B8] Kim S. Zhu Y. Romitti P. A. Fox D. J. Sheehan D. W. Valdez R. (2017). Associations between timing of corticosteroid treatment initiation and clinical outcomes in Duchenne muscular dystrophy. *Neuromuscul. Disord.* 27 730–737. 10.1016/j.nmd.2017.05.019 28645460 PMC5824693

[B9] Koeks Z. Bladen C. L. Salgado D. Van Zwet E. Pogoryelova O. McMacken G. (2017). Clinical outcomes in Duchenne muscular dystrophy: A study of 5345 patients from the TREAT-NMD DMD global database. *J. Neuromuscul. Dis.* 4 293–306. 10.3233/JND-170280 29125504 PMC5701764

[B10] Kong X. Zhong X. Liu L. Cui S. Yang Y. Kong L. (2019). Genetic analysis of 1051 Chinese families with Duchenne/Becker muscular dystrophy. *BMC Med. Genet.* 20:139. 10.1186/s12881-019-0873-0 31412794 PMC6694523

[B11] Liang W. C. Wang C. H. Chou P. C. Chen W. Z. Jong Y. J. (2018). The natural history of the patients with Duchenne muscular dystrophy in Taiwan: A medical center experience. *Pediatr. Neonatol.* 59 176–183. 10.1016/j.pedneo.2017.02.004 28903883

[B12] Niks E. H. Aartsma-Rus A. (2017). Exon skipping: A first in class strategy for Duchenne muscular dystrophy. *Expert Opin. Biol. Ther.* 17 225–236. 10.1080/14712598.2017.1271872 27936976

[B13] Okubo M. Noguchi S. Hayashi S. Nakamura H. Komaki H. Matsuo M. (2020). Exon skipping induced by nonsense/frameshift mutations in DMD gene results in Becker muscular dystrophy. *Hum. Genet.* 139 247–255. 10.1007/s00439-019-02107-4 31919629 PMC6981323

[B14] Pascual-Morena C. Martínez-Vizcaíno V. Saz-Lara A. López-Gil J. F. Fernández-Bravo-Rodrigo J. Cavero-Redondo I. (2022). Epileptic disorders in Becker and Duchenne muscular dystrophies: A systematic review and meta-analysis. *J. Neurol.* 269 3461–3469. 10.1007/s00415-022-11040-y 35229191

[B15] Ramesh V. Mountford R. Kingston H. M. Kelsey A. Noronha M. J. Clarke M. A. (1993). Occurrence of Duchenne dystrophy in Klinefelter’s syndrome. *Arch. Dis. Child.* 69 453–454. 10.1136/adc.69.4.453 8259881 PMC1029558

[B16] Rao M. V. Sindhav G. M. Mehta J. J. (2014). Duchenne/Becker muscular dystrophy: A report on clinical, biochemical, and genetic study in Gujarat population, India. *Ann. Indian Acad. Neurol.* 17:303. 10.4103/0972-2327.138508 25221400 PMC4162017

[B17] Santoro L. Pastore L. Rippa P. G. Orsini A. V. M. Del Giudice E. Vita G. (1998). Dystrophinopathy in a young boy with Klinefelter’s syndrome. *Muscle Nerve* 21 792–795. 10.1002/(sici)1097-4598(199806)21:6<792::aid-mus12>3.0.co;2-v 9585334

[B18] Sattenapalli N. C. Areti A. R. Gudhanti S. Kul U. Alavala R. R. (2022). Steroid versus non-steroid regimen in treating Duchenne muscular dystrophy: An observational study from South India. *Ind. J. Pharmaceut. Sci.* 84 80–86. 10.36468/pharmaceutical-sciences.898

[B19] Suthers G. K. Manson J. I. Stern L. M. Haan E. A. Mulley J. C. (1989). Becker muscular dystrophy (BMD) and Klinefelter’s syndrome: A possible cause of variable expression of BMD within a pedigree. *J. Med. Genet.* 26 251–254. 10.1136/jmg.26.4.251 2716035 PMC1017298

[B20] Thomas S. Conway K. M. Fapo O. Street N. Mathews K. D. Mann J. R. (2022). Time to diagnosis of Duchenne muscular dystrophy remains unchanged: Findings from the Muscular Dystrophy Surveillance, Tracking, and Research Network, 2000-2015. *Muscle Nerve* 66 193–197. 10.1002/mus.27532 35312090 PMC9308714

[B21] Tokgün O. Albuz B. Karagenç N. Erdoğan Ç Demiray A. Hakan A. (2022). A rare missense Duchenne muscular dystrophy gene variant in a family with muscular dystrophy from Turkey. *Eur. Res. J.* 8 225–231. 10.18621/eurj.944842

[B22] Torella A. Zanobio M. Zeuli R. del Vecchio Blanco F. Savarese M. Giugliano T. (2020). The position of nonsense mutations can predict the phenotype severity: A survey on the DMD gene. *PLoS One* 15:e0237803. 10.1371/journal.pone.0237803 32813700 PMC7437896

[B23] Tuffery-Giraud S. Béroud C. Leturcq F. Yaou R. B. Hamroun D. Michel-Calemard L. (2009). Genotype–phenotype analysis in 2,405 patients with a dystrophinopathy using the UMD–DMD database: A model of nationwide knowledgebase. *Hum. Mutat.* 30 934–945. 10.1002/humu.20976 19367636

[B24] Wang L. Xu M. Li H. He R. Lin J. Zhang C. (2019). Genotypes and phenotypes of DMD small mutations in Chinese patients with dystrophinopathies. *Front. Genet.* 10:114. 10.3389/fgene.2019.00114 30833962 PMC6388391

[B25] Wang Q. Yang X. Yan Y. Song N. Lin C. Jin C. (2011). Duchenne or Becker muscular dystrophy: A clinical, genetic and immunohistochemical study in China. *Neurol. Ind.* 59:797. 10.4103/0028-3886.91354 22234188

[B26] Wonkam-Tingang E. Nguefack S. Esterhuizen A. I Chelo D. Wonkam A. (2020). DMD-related muscular dystrophy in Cameroon: Clinical and genetic profiles. *Mol. Genet. Genomic Med.* 8:e1362. 10.1002/mgg3.1362 32543101 PMC7434738

[B27] Xu M. Fang F. Xu J. (2014). Rare combination of dystrophinopathy and Klinefelter’s syndrome in one patient. *Chin. J. Pediatr.* 52 548–551. 25224064

[B28] Zhang S. Qin D. Wu L. Li M. Song L. Wei C. (2021). Genotype characterization and delayed loss of ambulation by glucocorticoids in a large cohort of patients with Duchenne muscular dystrophy. *Orphanet J. Rare Dis.* 16 1–8. 10.1186/s13023-021-01837-x 33910603 PMC8082961

[B29] Zimowski J. G. Purzycka J. Pawelec M. Ozdarska K. Zaremba J. (2021). Small mutations in Duchenne/Becker muscular dystrophy in 164 unrelated Polish patients. *J. Appl. Genet.* 62 289–295. 10.1007/s13353-020-00605-0 33420945

